# Chromatographic analysis of age-related changes in mucosal serotonin transmission in the murine distal ileum

**DOI:** 10.1186/1752-153X-6-31

**Published:** 2012-04-11

**Authors:** Leena Parmar, Sara Fidalgo, Mark S Yeoman, Bhavik Anil Patel

**Affiliations:** 1 Centre for Biomedical and Health Sciences Research, University of Brighton, Brighton, BN2 4GJ, UK; 2 School of Pharmacy and Biomolecular Sciences, University of Brighton, Brighton, BN2 4GJ, UK

**Keywords:** Neurotransmission, Chromatography, Ileum, Serotonin, Ageing, Enterochromaffin cell

## Abstract

**Background:**

In the upper bowel, alterations in motility and absorption of key nutrients have been observed as part of the normal ageing process. Serotonin (5-HT) is a key signalling molecule in the gastrointestinal tract and is known to influence motility, however little is known of how the ageing process alters 5-HT signalling processes in the bowel.

**Results:**

An isocratic chromatographic method was able to detect all 5-HT precursors and metabolites. Using extracellular and intracellular sampling approaches, we were able to monitor all key parameters associated with the transmission process. There was no alteration in the levels of tryptophan and 5-HTP between 3 and 18 month old animals. There was a significant increase in the ratio of 5-HT:5-HTP and an increase in intracellular 5-HT between 3 and 18 month old animals suggesting an increase in 5-HT synthesis. There was also a significant increase in extracellular 5-HT with age, suggesting increased 5-HT release. There was an age-related decrease in the ratio of intracellular 5-HIAA:extracellular 5-HT, whilst the amount of 5-HIAA did not change with age. In the presence of an increase in extracellular 5-HT, the lack of an age-related change in 5-HIAA is suggestive of a decrease in re-uptake via the serotonin transporter (SERT).

**Conclusions:**

We have used intracellular and extracellular sampling to provide more insight into alterations in the neurotransmission process of 5-HT during normal ageing. We observed elevated 5-HT synthesis and release and a possible decrease in the activity of SERT. Taken together these changes lead to increased 5-HT availability and may alter motility function and could lead to the changes in adsorption observed in the elderly.

## Background

As part of the normal process of ageing, significant changes are known to arise within most of our organs and tissues, and the gastrointestinal (GI) tract has proved no exception [1,2]. The incidence of functional GI disorders increases in the upper bowel as we age, including absorptional disorders [2,3]. These disorders often lead to mal-absorption in the elderly which has been described as the ‘anorexia of ageing’ theory [4,5]. Although these disorders are well documented, little is known about the physiological changes that underlie these conditions. Age-related alterations have been observed in ion transporters, intestinal transit and the structures of the bowel [6-9], including an age-related reduction in neuronal density or number (mainly cholinergic neurons), particularly within the myenteric plexus [6,10,11].

Despite these studies, there appears to be an overall lack of functional data that has examined the effects of ageing on motility. Where studies exist in humans conflicting results have been observed [9]. This may be due to a combination of factors such as dietary alterations and co-morbidities as well as multiple drug therapies in the elderly, making it difficult to assess the impact that age-related changes have on these conditions. Animal models which are not influenced by such intrinsic and extrinsic factors have provided vital information about the impact ageing has on the GI tract in the absence of disease.

It is possible that the age-related changes may in part be due to alterations in signalling within the GI tract. Serotonin (5-HT), an important neurotransmitter and paracrine signalling molecule, is known to have a variety of biological functions. Specifically within the GI tract, it is largely responsible for the maintenance of both nutrition absorption and motility [12,13]. In addition, it is the intestinal mucosa that contains the largest amount of 5-HT in relation to the rest of the body, with 95% residing within enterochromaffin (EC) cells [14]. EC cells are thought to act as an important interface between the hostile environment of the gut lumen, and the neurons which are unable to penetrate it.

**Scheme 1:**
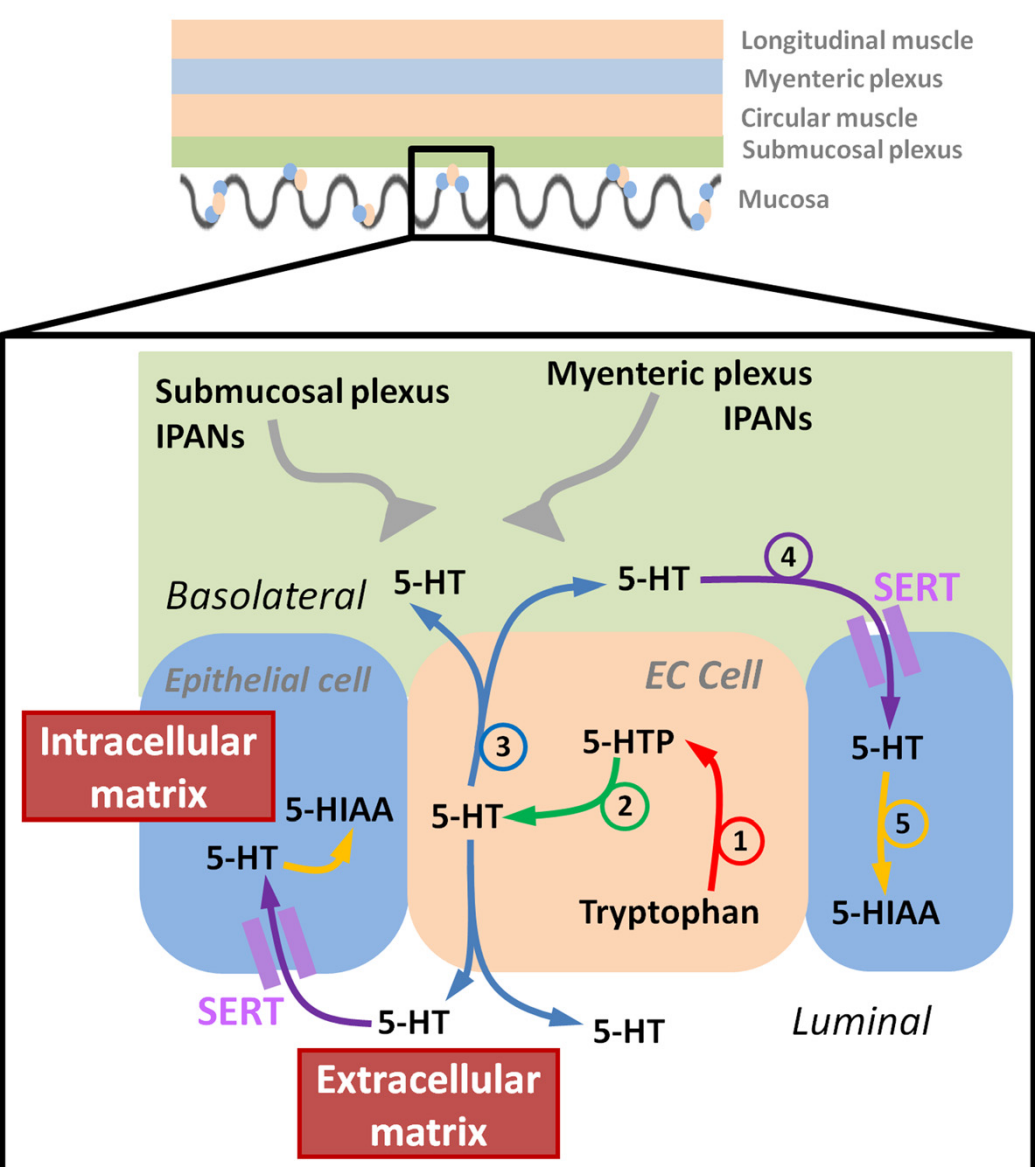
**5-HT signalling mechanism from enterochromaffin cells located within the lumen of the ileum.***Top* – Side view of the different layers that comprise the intestinal wall. The outer layer is the longitudinal muscle, followed by the myenteric plexus. A layer of circular muscle is next, followed by the submucosal plexus. The innermost layer is comprised of the mucosa where EC cells and epithelial cells are located along the villi. *Bottom* – Diagram of a single EC cell (centre) with two epithelial cells positioned either side. Steps 1–5 are the processes involved in 5-HT neurotransmission. The beginning of the synthesis pathway for 5-HT is represented by step 1, where 5-Hydroxytryptophan (5-HTP) is synthesised from tryptophan by the rate limiting enzyme tryptophan hydroxylase-1 (TpH-1). The synthesis of 5-HT from 5-HTP by the enzyme L-amino acid decarboxylase (L-AADC) is represented by step 2. The release of 5-HT into the extracellular matrix is represented by step 3, where it can bind to corresponding 5-HT receptors on intrinsic primary afferent neurons (IPANs) within the submucosal and myenteric plexus. The mechanism by which 5-HT is inactivated is represented by step 4, where 5-HT is transported into epithelial cells via the serotonin reuptake transporter (SERT). The final step in neurotransmission, represented by step 5 involves the metabolism of 5-HT into 5-hydroxyindole acetic acid (5-HIAA) by the enzyme monoamine oxidase A (MAO_A_).

Scheme 1 shows the process of 5-HT signalling from the EC cell. The enzyme tryptophan hydroxylase-1 (TpH-1) synthesises 5-hydroxytryptophan (5-HTP) from tryptophan (step 1). 5-HTP is then converted to 5-HT by the enzyme L-amino acid decarboxylase (L-AADC, step 2). In response to mechanical and/or chemical stimulation of the mucosa, intracellular levels of Ca^2+^ are increased in EC cells, which in turn results in the release of 5-HT into the extracellular matrix (step 3). 5-HT then enters the inner walls of the GI tract, where it binds to 5-HT receptors on intrinsic primary afferent neurons (IPANs) within the submucosal and myenteric plexus, which in turn activates interneurons and motor neurons within the enteric circuitry to drive the peristaltic reflex [13,15]. The majority of released 5-HT is cleared into neighbouring epithelial cells that surround the EC cell via the 5-HT transporter (SERT, step 4). Following clearance, 5-HT is metabolised to 5-hydroxyindole acetic acid (5-HIAA) by the enzyme monoamine oxidase A (MAO_A_, step 5). Moreover, the activity of SERT is thought to be an important determinant of both strength and duration of excitatory signals transmitted by 5-HT from the EC cell.

Given the important role the EC cell plays within the GI tract, it is understandable that the measurement of 5-HT, its precursors and metabolites should be fully monitored during normal ageing. Much work has been carried out on how components of the 5-HT signalling mechanism are altered in various disease states, although very few studies have fully elucidated changes in 5-HT signalling from EC cells [16-18]. This is mainly due to the limitations of the methods utilised and the assumptions that are placed on the resultant experimental data to explain observations. For example, studies that have utilised electroanalytical techniques, can only provide insight into levels of 5-HT overflow, which can be used to gain some insight into release and clearance of 5-HT, but cannot provide insight into the regulation of 5-HT synthesis or metabolism [19-21].

Despite the various techniques available to assess components of 5-HT signalling such as electrochemical sensors [22-25], enzyme-linked immunosorbent assay (ELISA) [26], fluorescence [27] and immunohistochemistry (IHC) [16,28], their uses remain limited as only one or two parameters involved in the process of 5-HT neurotransmission can be measured. However, in contrast, high performance liquid chromatography (HPLC) [18,29,30] is capable of measuring all parameters noted in Scheme 1. Within the field of gastroenterology, HPLC has been predominantly used to measure levels of analytes in tissue homogenates [18,28,30] and hence only intracellular neurochemicals are monitored, meaning the release of 5-HT from EC cells is entirely missed. On the other hand, there are methods such as microdialysis [31,32] that only measure extracellular levels of 5-HT and so intracellular neurochemicals are not taken into account.

In this study, we investigated murine distal ileum tissue samples from two different age groups (3 and 18 months). We discuss how the two methods of sampling can allow for all processes of 5-HT neurotransmission to be monitored. Finally, in relation to Scheme 1, we highlight the key age-related changes observed within the 5-HT neurotransmission mechanism and discuss their possible connections with the prevalence of dysmotility in the elderly.

## Results and discussion

### Intracellular and extracellular sampling

**Figure 1:**
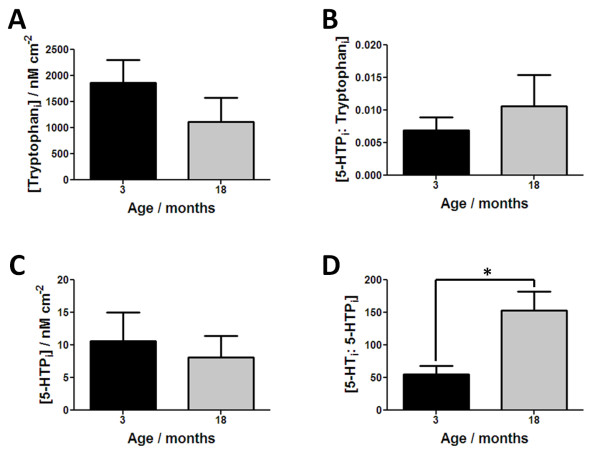
**Chromatographic responses in 3 month old ileum tissue, showing traces from intracellular (I) and extracellular (E) sampling.** Inset: shows the presence of the 5-HT peak from the extracellular sample. Responses of the standards (S) for the four neurochemicals are also shown. Solutes: 1–5-HTP, 2–5-HT, 3–tryptophan and 4–5-HIAA.

Figure 1 shows an example of a chromatogram from samples obtained using intracellular and extracellular sampling approaches. All the neurochemicals involved in the synthesis and metabolism of 5-HT can be monitored in the intracellular sample, however only 5-HT was observed in the extracellular sample. This is expected as shown in Scheme 1 only 5-HT is released from the EC cell and thus can be monitored. At present the majority of assays studying the efficacy of the transmission process rely on monitoring homogenised tissue samples, which can only monitor intracellular components [18,28,30]. Therefore assumptions about the transmission mechanism are being made based on what can be monitored and thus for example intracellular 5-HT is often used as a marker of release, even if only a fraction of this is actually released [33,34].

Utilising both intracellular and extracellular sampling strategies, ratios and levels of important signalling molecules can be monitored which accurately reflect on the process of transmission providing more insight than conventional approaches.

### Alterations in serotonin signalling

**Figure 2:**
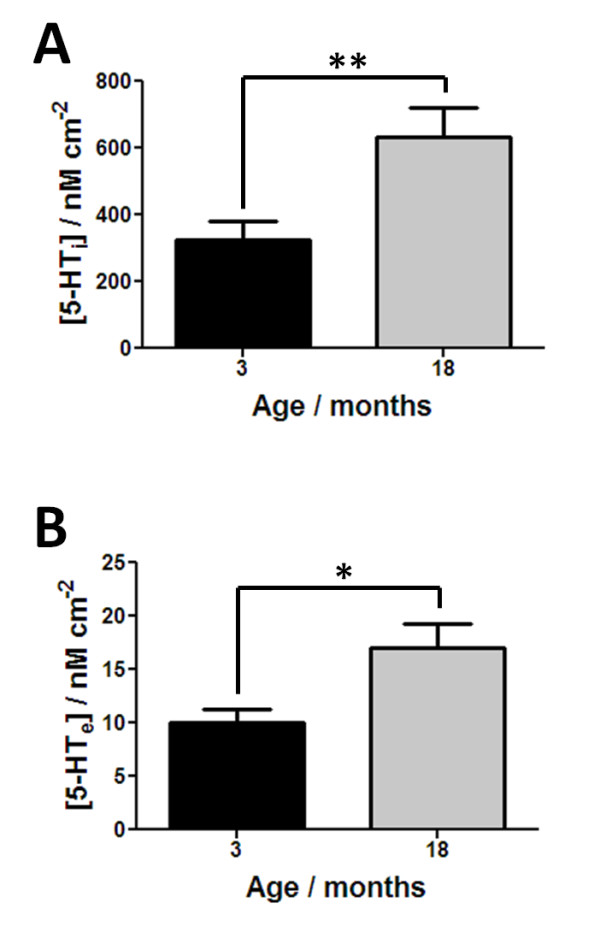
**Results showing factors associated with synthesis of 5-HT.** In **(a)** responses for intracellular tryptophan, **(b)** shows the ratio of 5-HTP: tryptophan, **(c)** shows levels of intracellular 5-HTP and **(d)** shows the ratio of 5-HT_i_: 5-HTP_i_. All results shown as mean ± S.E.M., where n = 7 and *p < 0.05.

Figure 2 shows how the levels of the components associated with the synthesis of 5-HT change with age. All these components were monitored from the intracellular sampling assay as they are formed and stored within the EC cell. There was no change in the amount of tryptophan between 3 and 18 month old tissue samples (Figure 2A, n = 7, *p* = 0.262). Similarly there was no significant difference in the level of the intermediate 5-HTP (Figure 2C, n = 7, *p* = 0.667), which resulted in no difference in the ratio of 5-HTP:tryptophan (Figure 2B, n = 7, *p* = 0.721) between age groups. This suggests that the activity of the enzyme tryptophan hydroxylase-1 is not altered between 3 and 18 month old animals. There is however a significant increase in the ratio of 5-HT:5-HTP between 3 and 18 month old animals (Figure 2D, n = 7, *p* < 0.05). This suggests that there is either an increase in activity or expression of the enzyme L-amino acid decarboxylase and thus an age-related increase in the synthesis of 5-HT. Other studies in various animal models have shown either increases or no changes in the levels of L-amino acid decarboxylase with age using biochemical and imaging approaches [35-37].

**Figure 3:**
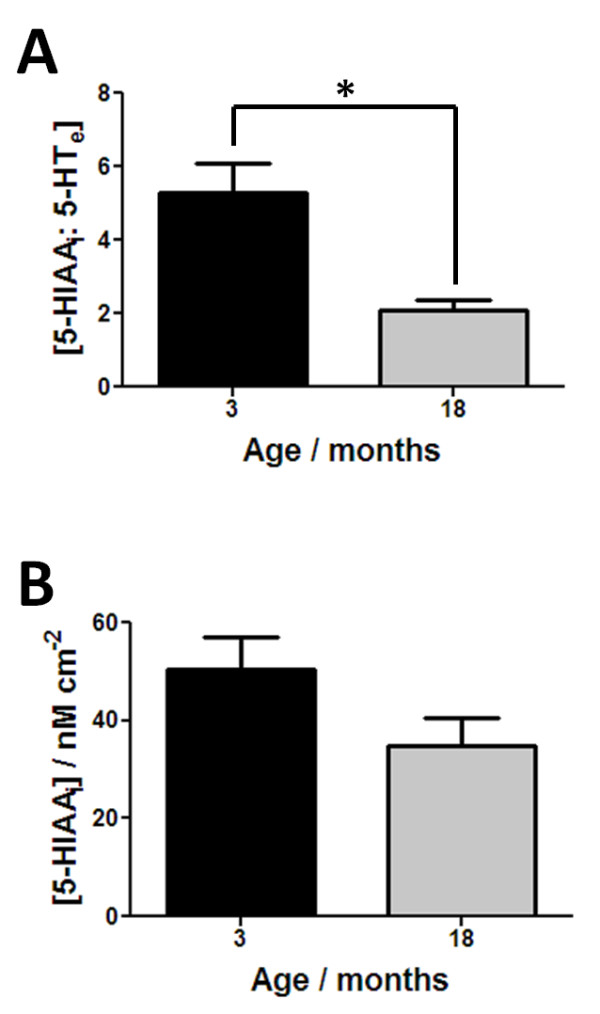
**Age-related changes in the release of 5-HT.** In **(a)** responses for intracellular 5-HT are shown, whilst in **(b)** extracellular 5-HT responses are shown. All results shown as mean ± S.E.M., where n = 6–7, *p < 0.05 and **p < 0.01.

There was a significant increase in intracellular serotonin (5-HT_i_) with age as shown in Figure 3A (n = 7, *p* < 0.01). This increase in 5-HT_i_ can be explained by a variety of factors, as it can be influenced by three factors: (i) synthesis from 5-HTP, (ii) amount released to the extracellular matrix, (iii) the amount of 5-HT repackaged for release following clearance via SERT and/or (iv) increase in the number of EC cells. Figure 3B shows that extracellular serotonin (5-HT_e_) significantly increases with age (n = 6–7, *p* < 0.05). This increase suggests that there is either an age-related increase in the release of 5-HT or a diminishment in the amount of activity of SERT. Other studies that have focused on investigating how 5-HT signalling changes with age have showed similar insights using chromatographic analysis. Although these studies have only measured intracellular 5-HT levels, increases have been observed in brain regions of various animal models [38-40].

**Figure 4:**
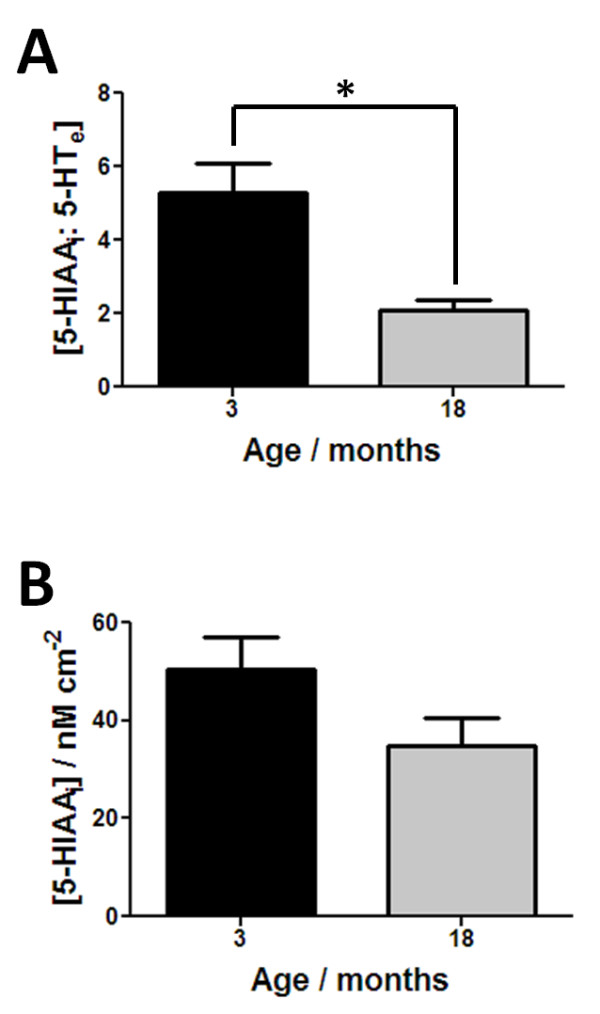
**Alterations in the clearance and metabolism of 5-HT between 3 and 18 month old animals.** In **(a)** responses for the ratio of intracellular 5-HIAA: extracellular 5-HT are shown, whilst in **(b)** intracellular 5-HIAA responses are shown. All results shown as mean ± S.E.M., where n = 6–7 and *p < 0.05.

Figure 4 shows the responses that reflect on the clearance and metabolism of 5-HT. There is a significant age-related decrease in the ratio of intracellular 5-HIAA: 5-HT_e_ (Figure 4A, n = 6–7, *p* < 0.05). From Scheme 1, this ratio provides insight into both the clearance and metabolism of 5-HT, as extracellular 5-HT is predominately cleared by SERT. Following this, the 5-HT is either repackaged for release or metabolised to 5-HIAA as denoted by step 5 in Scheme 1. The results in Figure 4A suggest that one or both of these processes may be diminished between 3 and 18 month old animals. To provide more insight, the effects of age on the level of intracellular 5-HIAA can be investigated. Figure 4B shows that there is no significant difference in the amount of intracellular 5-HIAA observed between both groups of animals. These findings suggest one of three possibilities: i) In the presence of an increase in extracellular 5-HT seen in the 18 month tissue, SERT activity is impaired so that a constant amount of 5-HT is being taken up by both age groups and metabolism/re-packageing remains constant, ii) More 5-HT is being taken up into the cell in the 18 month tissue but its metabolism to 5-HIAA is impaired and more is re-packaged into vesicles for release or iii) a combination of both scenarios is present. There is little or no evidence for MAO changing with increasing age but age-related decreases in the activity of SERT have previously been shown in the CNS of both mammalian and moluscan nervous systems [24,41]. In addition SERT expression has been shown to change in a wide variety of gastrointestinal disorders suggesting that this is the most plausible explanation for our data.

Overall we have observed an increase in the synthesis (Figure 2D) and release of 5-HT (Figure 3B). Our data also suggest an age-related decrease in the clearance of 5-HT (Figure 4A) in the distal ileum. The net effect of these age-related changes is an increase in the availability of 5-HT in 18 month old ileum. As 5-HT is well known to be associated with motility this increase in luminal 5-HT may have two potential effects: (i) increase in ileal motility due to over activation of cholinergic and nitregic pathways or (ii) desensitize 5-HT_3_ receptors present on myenteric and submucosal IPANs (shown in Scheme 1), leading to a disconnection to neuronal pathways in the bowel wall. Either is likely to alter the mixing within the lumen and effect adsorption. The latter could result in poor adsorption of nutrients potentially lead to mal-absorption. Such alteration in mucosal signalling mechanism may potentially explain the variety of age-related gastrointestinal conditions observed in the elderly and the onset of anorexia with ageing.

## Experimental

### Sample preparation

All animal experiments were carried out in compliance with the relevant laws and institution guidelines. C57BL/6 male mice (3 and 18 months old) were euthanized by cervical dislocation. Segments of distal ileum were removed and placed in oxygenated (95% O_2_ 5% CO_2_) Krebs buffer solution, pH 7.4 (composition in mM: 117 NaCl, 4.7 KCl, 2.5 CaCl_2_, 1.2 MgCl_2_, 1.2 NaH_2_PO_4_, 25 NaHCO_3_, and 11 glucose, all obtained from Sigmal Aldrich, UK) prior to sample preparation.

Two separate assays were carried out for both tissue segments in order to obtain concentrations of signalling molecules both within and released from the mucosa.

### Extracellular sampling assay

For the measurement of extracellular signalling molecules, 1 cm segments of distal ileum were transferred to a well containing Krebs buffer solution. The tissue samples were then cut along the mesenteric border and pinned out into a modified tissue culture plate forming a whole mount preparation. Fabrication of this modified well plate has been described previously [29]. Once the tissue section was pinned within the individual well, the tissue was washed twice using Krebs buffer to remove any waste or excised mucosa which may have arisen during tissue transfer. After this, all excess Krebs buffer was removed from the well leaving only the pinned out tissue. 1.5 mL of Krebs buffer (ambient temperature) was then added to the well and the tissue was left to stand for 30 min. After this time, a 250 μL aliquot of the buffer solution was taken and added to an Eppendorf tube containing 250 μL of 0.1M ice cold perchloric acid. The mixture was then centrifuged at 13,200 g at 4°C for 10 min. After centrifugation, the supernatant from the sample was filtered using Phenex RC membrane 0.2 μm 4 mm syringe filters (Phenomenex) to remove any particulates. These samples were then stored on ice prior to HPLC analysis. The pinned out tissue was then imaged for surface area measurements.

### Intracellular sampling assay

For the measurement of intracellular signalling molecules, 1 cm long segments of distal ileum were transferred to an inverted Petri dish over ice. The tissue sections were cut along the mesenteric border and laid flat with the mucosal layer uppermost. The tissue was rinsed with ice cold Krebs buffer to remove any waste from the tissue surface. Subsequently, the mucosal layer was scraped off containing the cells required for intracellular analysis using a scalpel from an approximately 1 cm^2^ segment of the tissue samples. The mucosal scraping was transferred to an Eppendorf tube and homogenised in 500 μL of ice cold perchloric acid for 2 minutes. The tissue samples were then centrifuged at 13,200 g at 4°C for 10 min. After centrifugation, the supernatant from the sample was filtered as above and stored on ice prior to HPLC analysis. The remaining tissue segment following mucosal scraping was pinned out to be imaged for surface area measurements.

### Chromatography

The HPLC apparatus consisted of a Jasco HPLC pump (Model: PU-980) and Rheodyne manual injector equipped with a 20 μl loop. A Kinetic® ODS 2.6 μm 100 mm x 2.1 mm i.d. analytical column with a KrudKatcher™ Ultra in-line filter (Phenomenex®, Macclesfield, UK) was employed. The HPLC system was used in a completely isocratic mode for the detection of the signalling molecules. The HPLC system was run at a flow rate of 100 μL min^-1^ and an injected sample volume of 20 μL was used. CHI1001A potentiostat (CH Instruments, Austin, TX, USA) was used to control the detector voltage and record the current. A 3 mm glassy carbon electrode (flow cell, BAS) served as the working electrode and was used with a Ag|AgCl reference electrode and a stainless steel block as the auxiliary electrode. Amperometric recordings were carried out, where the working electrode was set at a potential of +850 mV vs Ag|AgCl reference electrode. Control and data collection/processing were handled through the CHI1001A software.

The stock buffer for the mobile phase was comprised of the following: 0.1 M sodium acetate, 0.1 M citric acid and 27 μM disodium ethylene-diamine-tetra-acetate (EDTA) dissolved in 1 L of deionised water. This was then buffered to pH 3.0. The mobile phase was prepared with the stock buffer mixed with methanol in the ratio of 8:2 (v/v) and degassed after mixing.

### Standards and accuracy

Standard solutions were prepared from 500 μM stock standards of each analyte and were made up in 0.1 M perchloric acid (Σ). Each of the standard solutions were prepared on the day of analysis and stored at 4°C prior to injection. A calibration plot was obtained by running individually, a range of concentrations of 5-HTP, 5-HT, tryptophan and 5-HIAA (all obtained from Sigma Aldrich). Concentrations investigated were from the range of 0.1 -20 μM for 5-HT and 0.01-10 μM for 5-HTP, tryptophan and 5-HIAA. The peak areas obtained from chromatographic analysis of all injected samples were converted to concentrations using the calibration responses of the neurochemical standards.

### Data analysis and interpretation

As the concentration of the neurochemicals was analysed in both intracellular and extracellular conditions, a normalisation method was required that could be utilised in both approaches. We have utilised normalisation by tissue area using pixel counting algorithms as this can be utilised for both sampling approaches. The tissue area calculation by pixel counting algorithm is widely used in a variety of scientific fields [42,43]. The process of data normalisation utilised within this study has been described elsewhere [44]. Briefly, following chromatographic analysis for intracellular and extracellular levels of the neurochemicals, the pinned out tissue sections were photographed using a digital camera (7 megapixel) and the resultant image was analysed to calculate the surface area in pixels using IMAGE J. From the intracellular samples the area of the removed mucosal samples were used, whilst for the extracellular samples the total area of the tissue was analysed. Once all intracellular and extracellular images were converted to surface area in cm^2^, the concentration data was normalised to the surface area, to allow for comparison between both sampling assays. All data are shown as mean ± standard error of the mean (S.E.M) and *n* refers to the number of single tissue samples per animal included within the data set. Concentrations of individual neurochemicals were compared between 3 and 18 month old animals using the student *t*-test, whilst ratios of neurochemicals were analysed using the Mann Whitney test.

## Conclusions

We have utilised two sampling approaches which provide more suitable markers to study the full process of 5-HT signalling using chromatography. We have shown that age-related changes in 5-HT signalling occur between 3 and 18 month old animals. Increased synthesis and release coupled with reduced clearance were observed which ultimately leads to increased 5-HT availability within the ileum. Such increases in 5-HT may lead to altered motility which may underlie age-related GI disorders in the upper bowel.

## Competing interests

The authors declare that they have no competing interests.

## Authors’ contributions

LP carried out the experiment work, participated in data collection and manuscript drafting. SF carried out experimental work and assisted in data analysis. MSY assisted in data analysis, modified the text and provided funding for the study. BAP proposed, co-ordinated and supervised the study, participated in data analysis and drafted the manuscript. All authors read and approved the final manuscript.
